# Differential Expression of miRNA-223 in Coronary In-Stent Restenosis

**DOI:** 10.3390/jcm11030849

**Published:** 2022-02-06

**Authors:** Shiva Ganjali, Seyed Hamid Aghaee-Bakhtiari, Željko Reiner, Amirhossein Sahebkar

**Affiliations:** 1 Department of Medical Biotechnology and Nanotechnology, Faculty of Medicine, Mashhad University of Medical Sciences, Mashhad 9177948564, Iran; ganjalis@yandex.com; 2 Bioinformatics Research Group, Mashhad University of Medical Sciences, Mashhad 9177948564, Iran; bakhtiarisha@mailfa.com; 3 Department of Internal Medicine, School of Medicine, University Hospital Center Zagreb, University of Zagreb, 10000 Zagreb, Croatia; 4 Applied Biomedical Research Center, Mashhad University of Medical Sciences, Mashhad 9177948564, Iran; 5 Biotechnology Research Center, Pharmaceutical Technology Institute, Mashhad University of Medical Sciences, Mashhad 9177948954, Iran; 6 School of Medicine, The University of Western Australia, Perth, WA 6009, Australia; 7 Department of Biotechnology, School of Pharmacy, Mashhad University of Medical Sciences, Mashhad 9177948954, Iran

**Keywords:** in-stent restenosis, HDL-associated miRNA, miRNA-223

## Abstract

Objective: In-stent restenosis (ISR) is an unfavorable complication that occurs in patients after coronary stenting. Despite the progress with advent of modern DES and new antiplatelet agents, restenosis still hampers PCI short- and long-term results. The aim of this study was to investigate whether circulating miRNA-223, which is associated with HDL particles and involved in cholesterol efflux pathway, have diagnostic capability for determining ISR. Methods: This case–control study comprised 21 ISR and 26 NISR patients. The level of miRNA-223 expression was evaluated by TaqMan Real-Time PCR, quantified by the comparative method (fold change) and normalized to U6 expression. Results: Patients in ISR and NISR groups were not different in terms of demographic, clinical, and biochemical parameters, except that the percentage of patients who had DES was significantly greater in the NISR group (88.9%) in comparison with the ISR group (50%). The serum expression of miRNA-223 in ISR patients was 3.277 ± 0.9 times greater than that in NISR group (*p* = 0.016). In addition, the results of binary logistic regression demonstrated that the high level of serum miRNA-223 was strongly and positively associated with the ISR risk (OR: 17.818, 95% CI: 1.115–284.623, *p* = 0.042) after adjustment for age, sex, HDL-C, LDL-C, FBS, and statin consumption. Conclusion: Elevated serum level of miRNA-223 might be helpful in predicting the occurrence of ISR. Further confirmation in future large-scale studies is warranted.

## 1. Introduction

According to the World Health Organization (WHO) report in 2020, the main global reason of death in 2019 (16% of total death) is associated with ischemic heart disease (IHD), which is caused by atherosclerosis [1,2]. The percutaneous coronary intervention (PCI) with stent implantation is one of the most effective methods to restore coronary blood flow at atherosclerosis [3,4,5]. However, tissue damage attributed to stent placement may contribute to increase oxidative stress and inflammatory responses, platelet aggregation, fibrin deposition, stimulate the proliferation, migration, and apoptosis of vascular smooth muscle cells (VSMC) within the stent area, leading to the neointimal formation and finally the occurrence of in-stent restenosis (ISR) [4,5,6,7,8,9].

Although the antiplatelet regimes and drug-eluting stents (DES) are widely used to drastically reduce neo-atherosclerosis and neointimal hyperplasia, ISR complication still remains a clinical drawback occurring in patients who have undergone stent implantation [10,11,12,13]. Therefore, it is crucially important to find a sensitive and reliable biomarker in order to distinguish patients who are at a higher risk of ISR so that the right perspective can be provided on choosing appropriate prevention or even treatment strategies for these patients. Some evidence has suggested a potential role for miRNAs in predicting risk of cardiovascular diseases (CVDs) [14,15,16,17].

MicroRNAs (miRNAs) are small (18–22 nucleotides), single-stranded, and non-coding RNAs involved in regulating the expression of genes at the post-transcriptional level, affecting a wide range of pathophysiological processes including heart diseases [18,19,20]. MiRNAs are abundantly expressed in vascular tissues [21] and in a complex within the RNA-induced silencing complex (RISC) could induce translational inhibition and/or degradation of messenger RNA (mRNA) by binding to the its 3′-UTR [22] and modulate the expression of anti-pro-angiogenic genes [23,24,25]. MiRNAs could play an important role in different functions of vascular cells, such as cell differentiation, contraction, migration, proliferation, and inflammation that are involved in angiogenesis, neointimal formation, and lipid metabolism. All these phenomena occur in vascular destruction and restenosis [20,21,26]. Moreover, miRNAs by modulation of NF-KB [27] as a potential regulator of immunity, inflammation, cell survival, differentiation, and proliferation could affect many CVDs such as atherosclerosis [28]. In addition to the miRNAs involved in the vascular dysfunction [29,30], several studies have indicated the potential of miRNAs associated with lipids and lipoproteins metabolism, especially those are involved in the cholesterol efflux pathway, a key step of reverse cholesterol transport (RCT), as biomarkers for atherosclerosis [31,32,33,34,35].

Since the number of patients who undergo stent implantation is constantly increasing, it is important to diagnose patients who are at increased risk of ISR complication in order to prevent the mortality and morbidity in these patients. Therefore, this study aimed to evaluate the diagnostic importance of miRNA-223, an HDL-associated miRNA, as potential molecular marker for ISR, in order to improve the diagnosis of patients who are at increased risk.

## 2. Materials and Methods

### 2.1. Study Subjects

In this case–control study, 47 unrelated Iranian patients (18–75 years old) with a history of coronary stent implantation were selected on the basis of the second angiographic results at least 30 days after intervention. Patients who had >50% and <50% stenosis within the stent were placed into the in-stent restenosis (ISR; *n* = 21) and non-ISR (NISR; *n* = 26) groups, respectively. The study protocol was approved by the Ethics Committee of the Mashhad University of Medical Sciences and written informed consent was obtained from all participants. The clinical data including gender, age, past history of diabetes mellitus (DM), hypertension (HTN), dyslipidemia, smoking, medication, and duration between coronary stenting and subsequent angiography because of chest pain or equivalent symptoms were also collected from medical records. The exclusion criteria were primary PCI, positive troponin, restenosis in the first month after angioplasty due to thrombosis, autoimmune disorder, active cancer, thrombophilia, or chronic kidney disease.

### 2.2. Serum Preparation

Peripheral femoral or brachial blood was drawn right after entering the catheter and before starting angiographic procedure into tube with no anticoagulant. Serum was separated by centrifugation of the blood for 20 min at a relative centrifugal force of 1000 (recommended by manufacturer) and then stored at –80 °C prior to analysis.

### 2.3. Biochemical Measurements

Evaluation of fasting blood glucose (FBG), triglycerides (TG), total cholesterol (TC), and high-density lipoprotein cholesterol (HDL-C) were performed by Pars Azmoon kits (Tehran, Iran) on a BT-3000 auto-analyzer (Rome, Italy). Low-density lipoprotein cholesterol (LDL-C) was calculated using the Friedewald formula. Hs-CRP levels was also measured using a Biosystems assay kit on a BT-3000 auto-analyzer.

### 2.4. Serum RNA Extraction

In order to evaluate miRNA-223 expression, we extracted total RNA from 250 μL of the serum using the RNX-Plus (SINACLON, RN7713C/EX6101, Karaj, Iran) kit according to the manufacturer’s protocol with some modifications such as increasing the time of incubation and centrifugation to obtain the highest amount of miRNAs in the samples. The quantity and quality of the extracted RNAs was assessed using NanoDrop 2000 (Thermo, Wilmington, DE, USA), and samples with absorbance of 1.8–2 at 260/280 nm were used for miRNA specific cDNA synthesis.

### 2.5. Primer Design

The sequences of miRNA-223 and also U6 snRNA as an internal control were taken from the miRBase database. Then, RT stem loop, forward and reverse primers, and probes were designed specifically for miRNA-223 and internal control using AlleleID software (sequences are shown in Table 1). Oligo7 and GeneRunner software were also used to check the formation of secondary structures, including hairpin, homodimer, and heterodimer. Finally, the designed primers were blasted in NCBI to check the accuracy and specificity.

### 2.6. cDNA Synthesis and qRT-PCR

The reverse transcription was performed via first Strand cDNA Synthesis Kit (Yekta Tajhiz, Tehran, Iran, Cat No. YT4500) according to the manufacturer’s protocol. A total of 5 μg of total RNA with specific RT stem loop primer (Table 1) was used for cDNA synthesis via the thermocycler device for 5 min at 70 °C, 60 min at 42 °C, and 10 min at 70 °C. Synthetized cDNA was stored at −20 °C quantitative real-time PCR (qRT-PCR) analysis. Finally, TaqMan qPCR method was run in Light Cycler 96 instrument (Roche Diagnostics, Mannheim, Germany) using a 2× qPCR Master Mix (Ampliqon RealQ Plus Probe-Without Rox). All reactions were performed in duplicate (each 25 μL qPCR reaction included: 1 μL cDNA, 12.5 μL master mix, 0.5 μL of each primer (10 pmol/μL), 0.4 μL of probe, and 10.1 μL nuclease-free water). The following program was used to run qPCR reactions: 10 min at 95 °C, followed by 45 cycles of 30 s at 95 °C and 60 s at 60 °C. The level of miRNA-223 expression was measured using the Ct (cycle threshold) value and quantified by the comparative (2^−ΔΔCT^) method (fold change) and normalized to U6 expression.

### 2.7. Statistical Analysis

All analyses were performed using SPSS software, version 11.5 (Chicago, IL, USA). Data were checked for normality using the Kolmogorov–Smirnov test. All data were normally distributed and are presented as mean ± standard error (SE), and changes in variables were distinguished by independent samples *t*-test between two groups (ISR and NISR). Categorical variables are presented as percentages and were compared between groups by a chi-squared analysis or Fisher’s exact test. Relative expression software tool (REST) was used to analyze the miRNA-223 fold change expression level in ISR group relative to NISR patients. A correlation between study parameters was assessed using the Pearson correlation coefficient, and binary logistic regression was used to estimate the association between study parameters with ISR (ref: NISR) after adjustment for stent type (model I), as well as age, sex, LDL-C, HDL-C, FBG, and statin consumption (model II). *p*-values less than 0.05 were considered statistically significant.

## 3. Results

### 3.1. Baseline Characteristics of Patients

A total of 21 ISR and 26 NISR patients were enrolled in this study. The mean ages of the patients in ISR and NISR groups were 59.5 ± 2.6 and 60.4 ± 2.1 years, respectively. During the first PCI, 71.9% all patients underwent DES implantation; however, about half of them (44.7%) had experienced ISR complication. ISR and NISR groups were not statistically different in terms of gender, being a smoker, DM, HTN, dyslipidemia, medication, SBP, DBP, height, weight, and ejection fraction. However, the percentage of patients who had DES was significantly greater in the NISR group (88.9%) in comparison with the ISR group (50%) (Table 2). In addition, the biochemical parameters including FBS, TC, LDL-C, HDL-C, and hs-CRP showed no significant differences between the studied groups; however, a high level of TG (*p* = 0.059) was observed in ISR patients related to NISR patients, which was not statistically significant (Table 3).

Furthermore, the results of binary logistic regression illustrated that dyslipidemia (OR: 12.975; 95% CI: 1.357–124.029, *p* = 0.026) and having bare metal stents (OR: 8.00; 95% CI: 1.316–48.645, *p* = 0.024) were significantly associated with ISR risk, and such an association remained significant, even after adjustment for stent type as a confounder variable.

### 3.2. Comparison of Serum miRNA-223 Expression between Studied Groups

**Figure 1:**
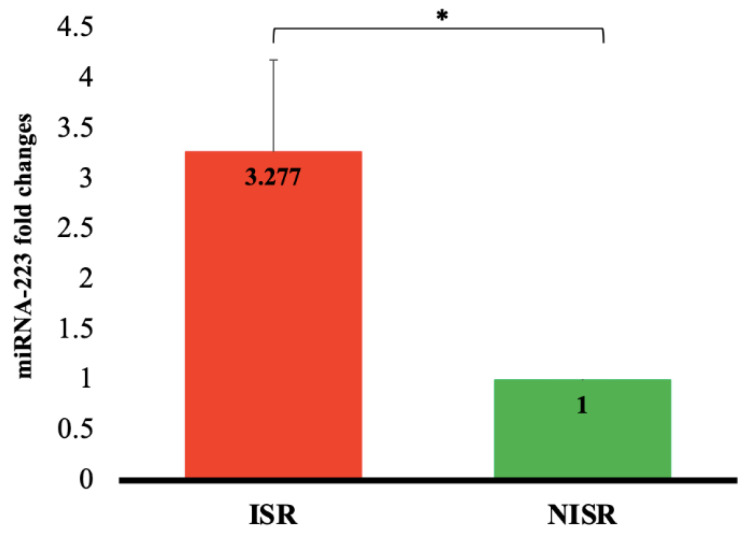
Serum miRNA-223 fold change expression in ISR patients related to NISR as a control group. Data are expressed as mean ± SE. * *p* < 0.05.

As outlined in Figure 1, the expression of miRNA-223 in ISR patients was 3.277 ± 0.9 times greater than that in NISR group (*p* = 0.016). In addition, the results of binary logistic regression demonstrated that upregulation of serum miRNA-223 was not associated with the ISR risk (OR: 3.077; 95% CI: 0.846–11.187, *p* = 0.088), even after adjustment for stent type as a confounder variable (model I), whereas when adjustment was performed for age, sex, HDL-C, LDL-C, FBS, and statin consumption (model II), the strong positive association was found between miRNA-223 upregulation and ISR risk (OR: 17.818; 95% CI: 1.115–284.623, *p* = 0.042) (Table 4).

In addition, the results of this study failed to show any correlation between miRNA-223 expression and other parameters such as age, LDL-C, HDL-C, TC, TG, FBS, BMI, and hs-CRP (data are not shown).

## 4. Discussion

It has been reported that both miRNAs [36] and lipoproteins such as HDL [37] could regulate many of the key processes involved in atherosclerosis [38]. Thus, the association between the expression of miRNAs related to the PBMCs [39], VSMCs [40,41,42,43], and vascular endothelial cells [44,45,46] with cardiovascular disease (CVD) and restenosis has been previously discussed [26,39,44]. However, to the best of our knowledge, there is no study that showed the serum expression level of miRNAs related to lipoproteins and lipid metabolism in ISR patients. The present study, for the first time, investigated the expression level of serum miRNA-223, an HDL-associated miRNA, in patients with ISR, finding that miRNA-223 was differentially overexpressed in these patients. In this regard, only one study in 2011 showed an increase in miRNA-233 expression in HDL obtained from individuals with familial hypercholesterolemia in comparison with healthy subjects [47]. In addition, in an experimental study the downregulation of miR-223 was indicated as a potential therapeutic approach to prevent restenosis after angioplasty [48].

MiRNA-223 is the most abundant miRNA carried by HDL particles [38]. This miRNA is secreted by myeloid cells and transported by HDL to the target cells such as endothelial cells, liver cells, SMCs, and monocytes [49]. It has been reported that the transcription and expression of miRNA-223 is increased followed by the accumulation of cholesterol in these cells as a feedback response leading to the inhibition of cholesterol synthesis in the cells, along with elevation of cholesterol efflux from the cells [50]. In addition, it was shown that miRNA-223 by inhibition of miRNA-33 could increase the ATP binding cassette A1 (ABCA1) expression, a key transporter of cholesterol efflux, and thus play an important role in cholesterol regulation and modulation and subsequently the RCT process [51]. MiRNA-223 also targets the 3’ region of the hepatic SR-BI gene and thus can influence the hepatic uptake of HDL-C as the last step in the RCT process [52,53].

Moreover, miRNA-223 is delivered to the endothelial cells via HDL and inhibits the expression of adhesion molecules such as ICAM1 on the surface of these cells, thereby causing the anti-inflammatory properties of HDL via restricting the infiltration of leukocytes into the endothelium [47].

Importantly, the results of our study illustrated that in patients who underwent coronary stent implantation, the serum level of miRNA-223 could not predict the ISR risk, even after adjustment for stent type as a confounder variable. On the other hand, when the CVD risk factors such as age, sex, HDL-C, LDL-C, FBS, and statin consumption were considered as confounder variables, elevated serum level of miRNA-223 could strongly and positively predict ISR risk.

Additionally, other variables such as de novo stenosis in other vessels, restenosis in more than one stent, size and brand of stent, and vessel caliber that might have an impact on ISR risk should consider as confounders in binary logistic regression. However, these data were not available for our patients, and this was the limitation of our study. On the other hand, the results of binary logistic regression analysis could be misleading in this case–control study with the small sample size, especially when adjusting for multiple variables was applied. This could be concluded by the wide range of CI. Thus, further confirmation in future large-scale studies, as well as in other ethnic groups is needed.

In conclusion, this study indicates that miRNA-223 overexpression by interfering with cholesterol metabolism as an important risk factor for CVD, as well as with endothelial cells activity, might be very important molecular marker in the diagnosis, prognosis, and even treatment of ISR patients.

## Figures and Tables

**Table 1:** Sequence of primers and probes used to evaluate the expression of miRNA-223.

miRNAs		Sequences	Product Length (bp)	Length (bp)
miRNA-223	RT stem loop	5′-GTCGTATGCAGAGCAGGGTCCGAGGTATTCGCACTGCATACGACTGGGGT-3′	50	69
	Forward	5′-CGCTGTCAGTTTGTCAAAT-3′	19	
	Reverse	5′-GAGCAGGGTCCGAGGT-3′	16	
	Probe	5′-FAM-CCCCAGTCGTATGCAGTGC-BHQ-1-3′	19	
U6 snRNA	RT stem loop	5′-GTCGTATGCAGAGCAGGGTCCGAGGTATTCGCACTGCATACGACAAAATATGG-3′	53	82
	Forward	5′-AAGGATGACACGCAAATTC-3′	19	
	Reverse	5′-GAGCAGGGTCCGAGGT-3′	16	
	Probe	5′-FAM-CGTTCCATATTTTGTCGTATGCAGT-BHQ-1-3′	25	

**Table 2:** Demographic and clinical characteristics of patients.

Variables		ISR (*n* = 21)	NISR (*n* = 26)	*p*-Value
Sex %	Male	52.4	50.0	0.871
	Female	47.6	50.0	
Age (y)		59.5 ± 2.6	60.4 ± 2.1	0.785
Height (cm)		161.5 ± 2.6	162.8 ± 2.0	0.678
Weight (kg)		69.9 ± 2.8	73.2 ± 3.5	0.492
BMI (kg/m^2^)		27.0 ± 0.9	27.5 ± 1.0	0.757
Smoker %		5.9	23.1	0.215
Dyslipidemia %		71.4	46.2	0.081
DM %		61.9	46.2	0.282
HTN %		71.4	65.4	0.659
Drugs consumption %	Statin	100.0	88.0	0.239
	Aspirin	90.5	84.0	0.257
	Clopidogrel	68.4	92.0	0.095
	Insulin	19.0	19.2	0.987
	Oral diabetic drugs	38.1	19.2	0.151
SBP (mmHg)		125.7 ± 3.6	121.8 ± 2.9	0.400
DBP (mmHg)		78.1 ± 2.4	74.8 ± 1.6	0.241
Stent type %	Bare metal	50.0 50.0	11.1 88.9	0.015 *
	Drug-eluting			
Stent number %	1	66.7	65.4	0.927
	>1	33.3	34.6	
Duration of stent implantation (month)		32.8 ± 5.9	22.4 ± 5.4	0.200
EF (%)		46.2 ± 2.9	45.2 ± 2.5	0.806

Data are expressed as mean ± SE or percentage; *: statistically significant (*p* < 0.05); BMI: body mass index; DM: diabetes mellitus; HTN: hypertension; SBP: systolic blood pressure; DBP; diastolic blood pressure. EF: ejection fraction.

**Table 3:** Comparison of biochemical parameters between studied groups.

Variables	ISR (*n* = 21)	NISR (*n* = 26)	*p*-Value
FBS (mg/dL)	155.6 ± 16.9	138.9 ± 15.7	0.473
TC (mg/dL)	145.6 ± 7.9	124.4 ± 8.7	0.084
TG (mg/dL)	144.8 ± 21.2	97.3 ± 11.8	0.059
HDL-C (mg/dL)	36.6 ± 1.6	34.1 ± 2.2	0.395
LDL-C (mg/dL)	81.7 ± 6.7	70.8 ± 6.4	0.252
hs-CRP (mg/L)	3.9 ± 0.8	4.6 ± 0.8	0.509

Data are expressed as mean ± SE, FBS: fasting blood sugar; TC: total cholesterol; TG: triglyceride; HDL-C: high-density lipoprotein cholesterol; LDL-C: low-density lipoprotein cholesterol; hs-CRP; high-sensitivity C-reactive protein.

**Table 4:** Binary logistic regression for miRNA-223 expression in relation with ISR (ref: NISR).

Variables	Unadjusted		Adjusted			
			Model I		Model II	
	OR (95% CI)	*p*-Value	OR (95% CI)	*p*-Value	OR (95% CI)	*p*-Value
miRNA-223 expression (FC)	3.077 (0.846–11.187)	0.088	1.885 (0.610–5.825)	0.271	17.818 (1.115–284.623)	0.042

FC: fold change; model I: adjusted for stent type; model II: adjusted for age, sex, HDL-C, LDL-C, FBS, and statin consumption.

## Data Availability

Data are available from the corresponding authors upon a reasonable request.
